# Modeling Infectious Bursal Disease Virus (IBDV) Antigenic Drift In Vitro

**DOI:** 10.3390/v15010130

**Published:** 2022-12-31

**Authors:** Amin S. Asfor, Vishwanatha R. A. P. Reddy, Salik Nazki, Joanna Urbaniec, Andrew J. Brodrick, Andrew J. Broadbent

**Affiliations:** 1 Birnaviruses Group, The Pirbright Institute, Ash Road, Woking GU24 0NF, UK; 2 Department of Comparative Biomedical Sciences, Section Infection and Immunity, School of Veterinary Medicine, Faculty of Health and Medical Sciences, University of Surrey, Guilford GU2 7AL, UK; 3 Pandemic Sciences Institute, Nuffield Department of Medicine, University of Oxford, Oxford OX3 7DQ, UK; 4 Department of Animal and Avian Sciences, College of Agriculture and Natural Resources, University of Maryland, College Park, MD 20742, USA

**Keywords:** infectious bursal disease virus (IBDV), antigenic drift, immune escape, escape mutant, hypervariable region, HVR

## Abstract

Infectious bursal disease virus (IBDV) vaccines do not induce sterilizing immunity, and vaccinated birds can become infected with field strains. Vaccine-induced immune selection pressure drives the evolution of antigenic drift variants that accumulate amino acid changes in the hypervariable region (HVR) of the VP2 capsid, which may lead to vaccine failures. However, there is a lack of information regarding how quickly mutations arise, and the relative contribution different residues make to immune escape. To model IBDV antigenic drift in vitro, we serially passaged a classical field strain belonging to genogroup A1 (F52/70) ten times, in triplicate, in the immortalized chicken B cell line, DT40, in the presence of sub-neutralizing concentrations of sera from birds inoculated with IBDV vaccine strain 2512, to generate escape mutants. This assay simulated a situation where classical strains may infect birds that have suboptimal vaccine-induced antibody responses. We then sequenced the HVR of the VP2 capsid at passage (P) 5 and 10 and compared the sequences to the parental virus (P0), and to the virus passaged in the presence of negative control chicken serum that lacked IBDV antibodies. Two escape mutants at P10 had the same mutations, D279Y and G281R, and a third had mutations S251I and D279N. Furthermore, at P5, the D279Y mutation was detectable, but the G281R mutation was not, indicating the mutations arose with different kinetics.

## 1. Introduction

Infectious bursal disease virus (IBDV) causes Gumboro disease in infected chickens, the severity of which varies depending on the strain. Infected birds may be asymptomatically infected, or they may suffer from a range of symptoms, with morbidity reaching 100% in birds infected with very virulent (vv) strains [1]. One of the hallmarks of infection is immunosuppression, which renders birds more susceptible to secondary infections and less responsive to vaccination programs against other diseases. Consequently, the economic significance of infection is due to morbidity and mortality caused by IBDV in its own right, and also due to immunosuppression [1]. IBDV belongs to the *Birnaviridae* family, and is non-enveloped with a genome comprised of double stranded RNA, which is divided into two segments, A and B. Segment B encodes VP1, which is the viral polymerase, and segment A encodes all other genes, including VP2, which forms the capsid, and is the main target of neutralizing antibody responses [1]. Within the VP2 molecule is a hypervariable region (HVR), between amino acids 220 to 330, which is under the most intense immune selection pressure that drives evolution at this site. Within the HVR, there are four hydrophilic loops (P-BC, P-DE, P-FG, and P-HI), which are thought to be the sites that the majority of neutralizing antibodies target. Mutations within the HVR can lead to alterations in virulence, tissue tropism, and antigenicity [2,3,4,5].

The cornerstone of IBDV control has been the use of vaccines, and multiple platforms are available, including live attenuated vaccines, inactivated vaccines, immune complex vaccines, and vectored vaccines based on the herpesvirus of turkeys (HVT) [6]. However, despite 50 years of vaccination, the disease remains endemic in almost every country in the world, and a significant threat to the global poultry industry. Vaccines aim to induce high levels of neutralizing antibodies in the serum, but they do not induce sterilizing immunity, meaning vaccinated flocks can become infected with wild type (wt) field strains that then can evolve. In addition vaccine failures are reported in the field, which have been associated with inappropriate vaccine scheduling and handling, leading to sub-optimal immune responses against circulating field strains [7]. This provides an ideal environment for the evolution of antigenic drift variants containing mutations in the HVR. However, our knowledge of which mutations are responsible for antibody escape is lacking, and the kinetics with which they arise remain poorly understood. To address this, we serially passaged a classical field strain belonging to genogroup A1 (F52/70) in the immortalized chicken B cell line, DT40, in the presence of sub-neutralizing concentrations of sera from birds inoculated with the vaccine strain 2512, to generate antibody escape mutants. We then sequenced the HVR at different passages to identify changes that contributed to immune escape.

## 2. Materials and Methods

### 2.1. Cell Lines, Viruses, and Antibodies

The chicken B-cell lymphoma cell-line, DT40 (ATCC, catalog number CRL-2111), was maintained in RPMI media supplemented with 10% heat-inactivated fetal bovine serum (FBS) (Sigma-Aldrich, Gillingham, UK), 1% l-glutamine (Sigma-Aldrich), 10% tryptose phosphate broth (Sigma-Aldrich), 1 mM sodium pyruvate (Sigma-Aldrich), and 50 mM beta-mercaptoethanol (Gibco) (complete DT40 media). The classical IBDV field strain F52/70 was a kind gift from Dr Nicolas Eterradossi (ANSES, Ploufragen, France). The virus was propagated in vivo by harvesting the bursa of Fabricius (BF) from experimentally inoculated chickens at 72 h post infection (hpi). Chickens were specific pathogen free (SPF) and were of the Rhode Island Red breed. The bursal material was pooled and homogenized in Vertrel XF (Sigma-Aldrich, Merck, Gillingham, UK), which separated into two phases. The upper phase was harvested and layered on top of a 30% sucrose solution and ultra-centrifuged at 27,000 rpm. The resulting pellet was resuspended in phosphate-buffered saline (PBS). The primary antibody used in this study was a mouse monoclonal antibody raised against IBDV VP3 protein (clone FC3) that was generated by Dr Kim Wark, is available through the UK Immunological Toolbox, and has been previously used in our lab [8,9,10]. The secondary antibody was a goat-anti-mouse monoclonal antibody conjugated to Alexafluor 488 (Invitrogen, Thermo Fisher Scientific, Horsham, UK). Anti-IBDV hyper-immune serum was obtained from Charles River (Frederick, Maryland, MD, USA). Briefly, SPF White Leghorn birds were repeatedly inoculated with antigen from IBDV vaccine strain 2512, and the serum was pooled, and lyophilized (anti-2512 serum). Negative control serum was obtained from Charles River, which consisted of serum from SPF White Leghorn birds that was pooled and lyophilized in the same way as the anti-2512 serum. The anti-2512 serum and the negative control serum were reconstituted in water, according to the manufacturer’s instructions.

### 2.2. Titration of IBDV in DT40 Cells

IBDV was diluted ten-fold in complete DT40 media in U-bottom 96-well plates (Falcon, Corning, UK), in quadruplicate, and DT40 cells were then added to diluted virus at 1 × 10^5^ cells/well. Cultures were incubated for 3 days, fixed in 4% paraformaldehyde solution (Sigma-Aldrich) for 20 min, permeabilized with a solution of 0.1% Triton X-100 (Sigma-Aldrich) for 10 min, and blocked with a 4% BSA solution for 60 min. The cells were then incubated with a primary mouse monoclonal antibody raised against the IBDV VP3 protein, diluted 1:100 in the blocking solution for 1 h at room temperature. VP3 was chosen as it was not subject to antibody selection pressure and so the monoclonal antibody consistently recognized this antigen. Cells were washed with PBS and incubated with a goat-anti-mouse secondary antibody conjugated to Alexa fluor 488 (Thermo Fisher Scientific) at a dilution of 1:500 in blocking solution for 1 h at room temperature in the dark. The cells were again washed and incubated for 10 min in a solution of 4′,6′-diamidino-2-phenylindole (DAPI) (Invitrogen, Thermo Fisher Scientific). Cells were imaged using a Leica DM IRB epifluorescence microscope. The highest dilution of the virus where 50% of the wells had a VP3 signal was considered as the end point, and the virus titer was determined from the tissue culture infectious dose-50 (TCID_50_), according to the method of Reed and Muench, and expressed as TCID_50_/mL [11].

### 2.3. Serial Dilution of Neutralizing Antibodies

Hyperimmune serum obtained from Charles River was heated at 56 °C for 30 minutes to inactivate complement factors, and serially diluted two-fold from 1:20. Diluted serum was incubated with 100 TCID_50_ of IBDV strain F52/70 for one hour at 37 °C, and the mixtures were incubated with 1 × 10^5^ DT40 cells in 96 well U-bottom Plates. Three days post-inoculation, cells were fixed and stained with an anti-IBDV VP3 antibody and a goat-anti-mouse secondary antibody conjugated to Alexafluor 488. Wells were scored as either positive or negative for IBDV antigen by immunofluorescence microscopy, and the highest dilution of the serum where there were VP3- positive cells was determined.

### 2.4. Generation of Escape Mutants

**Figure 1:**
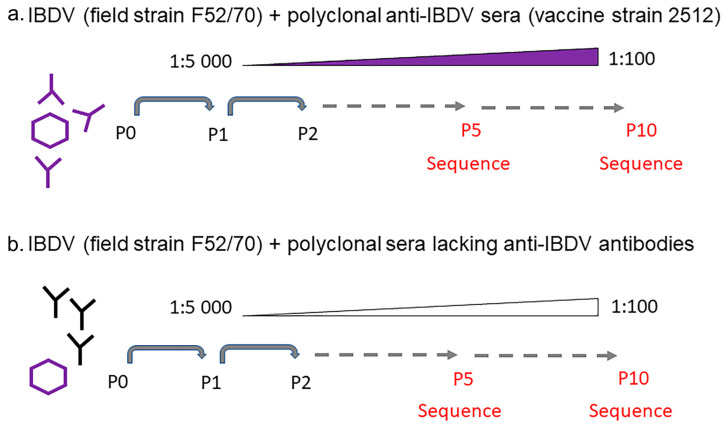
Generation of IBDV escape mutants. 100 TCID_50_ of IBDV strain F52/70 was mixed with 1:5000 of either (**a**) hyperimmune serum obtained from inoculating chickens with IBDV vaccine strain 2512, or with (**b**) negative control serum that did not contain any anti-IBDV antibodies. The mixture was incubated for 1 h at 37 °C, and added to DT40 cells, which were then incubated for 3 days at 37 °C, lysed, and the quantity of virus determined by a TCID_50_ assay. Then, virus at each passage was mixed with increasing concentrations of serum and passaged onto fresh DT40 cells. This protocol was repeated for ten passages. The sequences of the HVR of the viruses at passages 5 and 10 were determined by Sanger sequencing.

The highest dilution of the anti-2512 serum where there was still a detectable VP3 signal (1:5000), was mixed with 100 TCID_50_ of IBDV strain F52/70 and incubated for one hour at 37 °C. The mixtures were incubated with 1 × 10^5^ DT40 cells per well in 96 well U-bottom plates, in triplicate, for three days, whereupon the cells were lysed by three freeze–thaw cycles and the lysate clarified by centrifugation at 1250 rpm for 5 min. The quantity of virus in the clarified supernatant was determined by TCID_50_ assay, and the ability of the virus to escape neutralization was determined by conducting a neutralization assay. Then, virus at each passage was mixed with the lowest concentration of anti-2512 that permitted viral replication, incubated for 1 h at 37 °C, and 100 TCID_50_/well was passaged onto fresh DT40 cells. This protocol was repeated for 5 passages. Passage (P)1 = 1:5000 serum, P2 = 1:2500, P3 = 1:1250, P4 = 1:625, and P5 = 1:312 (Figure 1). At P5, a new batch of the anti-2512 hyperimmune sera was used, and the permissive titer was found to be 1:400, which was subsequently used in P6. As the immune pressure increased, the virus replicated less well, and the virus had to be re-passaged twice at the same concentration of serum before there was sufficient virus for the next passage: P6 = 1:400 serum, P7 = 1:400, P8 = 1:200, P9 = 1:200, and P10 = 1:100. As a negative control, F52/70 was passaged in the negative control chicken serum (Charles River) that did not contain anti-IBDV antibodies, used at the same dilutions as the positive serum.

### 2.5. Sequencing

RNA was extracted from the inoculum, and from cell lysates at passage 5 and 10 using an RNeasy kit (Qiagen, Manchester, UK), and reverse transcribed with Superscript III (Thermo Fisher Scientific) using a random primer, according to the manufacturer’s instructions. The sequences of HVRs of the rescued chimeric viruses were confirmed by using forward primer 5′-GCCCAGAGTCTACACCAT-3′ and reverse primer 5′-ATGGCTCCTGGGTCAAATCG-3′ (Integrated DNA Technologies, Leuven, Belgium) [12].

### 2.6. Bioinformatics Analysis of VP2 HVR

MEGA 6.0.6 was used to perform multiple-sequence alignments of the HVR sequences [13]. Amino acid identities of the HVR sequences were determined using the p-distance model.

### 2.7. Structural Modelling of Chimeric VP2 Molecules

The sequences of the VP2 genes from the wt F52/70 virus stock (passage 0), the F52/70 passaged in negative serum, and the escape mutants passaged in anti-2512 serum were translated in silico using SnapGene (version 6.0.2, GSL Biotech, San Diego, CA, USA), and the amino acid sequences were modelled using a modified version of AlphaFold v2.1.0 [14]. The models were then downloaded and processed using PyMol (version 2.5, Schrödinger, Cambridge, UK) to isolate the HVR and highlight residues that differed from wt F52/70.

### 2.8. Statistical Analysis

Neutralization titers were compared by one-way analysis of variance (ANOVA) with Tukey post hoc comparisons using GraphPad Prism version 7.01 (GraphPad Software, Inc., San Diego, CA, USA). Results were considered significantly different when *p* < 0.05.

### 2.9. Biosafety

To mitigate the risks of making IBDV escape mutants, we conducted our studies using the classical strain F52-70, as it is a representative classical strain that has circulated in vaccinated chicken flocks for years. Consequently, our study mimicked what already happens in nature. We also did not generate immune escape variants of vv strains. To prevent any environmental contamination with immune escape mutants, all virus work was carried out in a BSL2 laboratory, in a microbiological safety cabinet approved for BSL2 work. All personnel wore appropriate personal protective equipment (PPE), which consisted of a lab coat and nitrile gloves, and all waste was decontaminated with 1% Virkon for at least 10 min, which is known to inactivate IBDV, and solid waste was autoclaved prior to disposal, as per the Biological Agent Risk Assessment approved by The Pirbright Institute, UK.

## 3. Results

### 3.1. Serial Passage of F52/70 in Sub-Neutralizing Concentrations of Anti-2512 Serum Led to Immune Escape in DT40 Cells

**Figure 2:**
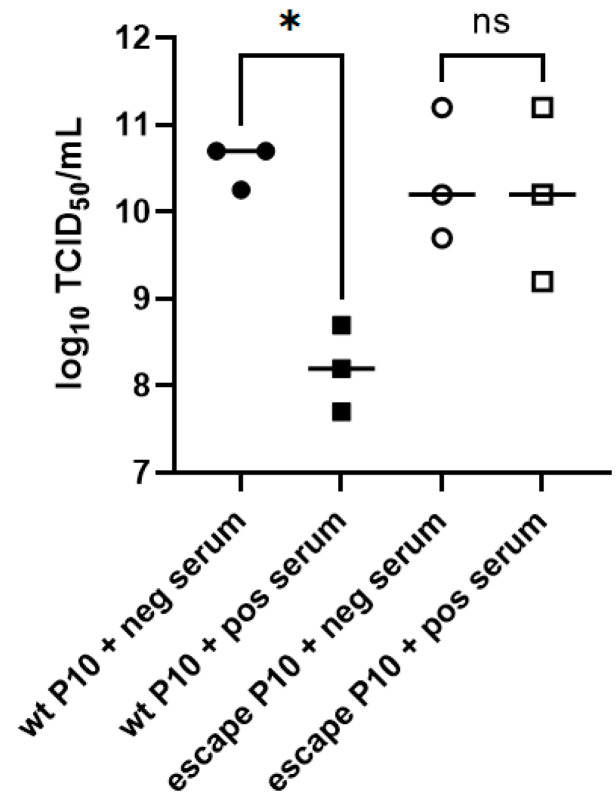
Passage of F52/70 in sub-neutralizing concentrations of anti-2512 serum led to immune escape in DT40 cells. The F52/70 virus that was passaged 10 times in serum lacking IBDV antibodies (wt P10), and the F52/70 virus that was passaged 10 times in anti-2512 serum to generate an escape mutant (escape P10), were titrated in triplicate in the presence of either negative control serum (neg serum) or anti-2512 serum (pos serum) at a dilution of 1:100. The virus titer was quantified, expressed as log_10_ TCID_50_/mL, and plotted on the y axis. A one-way ANOVA with Tukey post hoc comparisons was conducted, and results were considered significantly different when *p* < 0.05 (*), ns = “not significant”.

The wt classical F52/70 strain of IBDV was mixed with anti-2512 serum at the highest dilution that permitted viral replication (1:5000), the mixture was added to DT40 cells, and the cultures were incubated. Every three days, the cultures were passaged in increasing concentrations of anti-2512 serum to generate an escape mutant (Figure 1). Chicken serum that lacked antibodies against IBDV was used as a control (negative control serum). At passage (P) 10, the virus that was passaged in negative control serum (wt P10), or the virus that was passaged in the anti-2512 serum to generate an escape mutant (escape P10) were titrated in triplicate in the presence of negative control serum or anti-2512 serum (Figure 2). The wt and the escape P10 viruses replicated to an average of 10.55 (Standard Deviation (SD) 0.26) and 10.37 (SD 0.74) log_10_ TCID_50_/mL, respectively in the presence of negative control serum, and there was no significant difference between them (*p* = 0.98). In the presence of anti-2512 serum, the escape P10 virus replicated to a comparable titer (average,10.20 (SD 1.0) log_10_ TCID_50_/mL), whereas the wt P10 virus replicated to a significantly lower titer, 8.20 (SD 0.5) log_10_ TCID_50_/mL, *p* = 0.01. Taken together, these data demonstrated that we had successfully generated an escape mutant using our approach.

### 3.2. IBDV F52/70 Immune-Escape Mutants Had Mutations in the HVR

**Figure 3:**
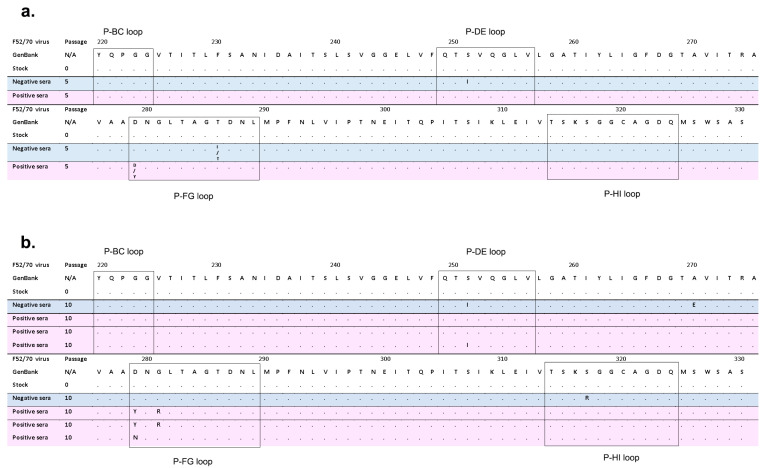
Alignment of the HVR amino acid sequence from IBDV strain F5/70 passaged in positive or negative serum. (**a**) The amino acid sequence of the VP2 HVR of the IBDV strain F52/70 that is in GenBank (Accession number AY321953) was aligned to the following sequences: the stock F52/70 virus at passage (P)0, the F52/70 virus that had been passaged 5 times in negative serum lacking anti-IBDV antibodies (Negative sera) (blue shading), and the F52/70 that had been passaged 5 times in increasing concentrations of anti-2512 serum (Positive sera) (pink shading). (**b**) The F52/70 GenBank sequence was aligned to the stock F52/70 virus at P0, the F52/70 virus at P10 in Negative sera (blue shading), and the three independently generated escape mutants comprised of the F52/70 virus at P10 in Positive serum (pink shading). Amino acids that were identical to the F52/70 GenBank sequence were labelled with a dot, and amino acids that were different from F52/70 were assigned their lettered code. The four hydrophilic loops within the HVR (loops P-BC, P-DE, P-FG, and P-HI) were boxed.

We sequenced the HVR of the viruses that had been passaged in either negative control serum (Negative sera), or anti-2512 serum (Positive sera) at P5 and P10, and compared the sequences to the parental virus (P0) (Figure 3). At P5, the virus passaged in anti-2512 serum had a mixed nucleotide chromatogram trace that translated to either aspartic acid (D), or tyrosine (Y) at amino acid position 279, whereas the P0 virus had D at this position (Figure 3a). At passage 10, the Y mutation had become fixed in the virus population and there was no longer any indication by Sanger sequencing of a D at this site (D279Y) (Figure 3b). Furthermore, there was a glycine (G) to arginine (R) mutation at amino acid 281 (G281R) at P10 that was not present at P0 or P5, indicating the mutations arose with different kinetics. In the virus that was passaged 5 or 10 times in negative control serum, we found the following mutations associated with DT40 cell adaptation at P10: serine to isoleucine at position 251 (S251I), alanine to glutamic acid at position 270 (A270E), and serine to arginine at position 317 (S317R) (Figure 3b). Of these mutations, S251I arose at P5, but the others were not detected at this passage (Figure 3a). We then generated two additional escape mutants independently, by the same method, and sequenced them at P10. We found that one of them contained the same D279Y and G281R mutations, and the other had mutations S251I, and aspartic acid to asparagine at position 279 (D279N) (Figure 3b).

### 3.3. IBDV F52/70 Immune-Escape Mutants Had Mutations on Both the Side and Axial Tip of the VP2 Molecule

**Figure 4:**
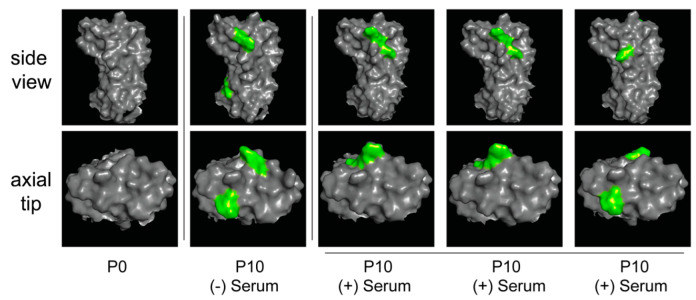
Structural modelling of the HVRs. The predicted structure of the HVR of VP2 of IBDV strain F52/70 was modelled using AlphaFold. Images were generated with PyMol, and the side view (upper panels) and end-on/axial tip view (lower panels) of each molecule were displayed. The HVR was depicted as solid grey, and the predicted structure of the parental F52/70 virus before passage (P0) was compared to the F52/70 virus that was passaged 10 times in serum that lacked IBDV antibodies (P10 (-) Serum), and the three escape mutants that were passaged 10 times in anti-2512 serum (P10 (+) Serum). Amino acid differences between the passaged viruses and the P0 HVR were highlighted in green. The P10 (-) Serum virus contained the S251I, A270E, and S317R mutations, the first and second P10 (+) Serum viruses contained the D279Y and G281R mutations, and the last P10 (+) Serum virus contained the S251I and D279N mutations.

We modelled the structure of the HVR of the parental F52/70 virus at P0, the virus passaged ten times in negative control serum ((-) Serum), and the three viruses passaged ten times in anti-2512 serum ((+) Serum), and we highlighted mutations that were present in the passaged viruses that were not present in the P0 virus (Figure 4). Mutations that arose following passage in both negative control serum, and anti-2512 serum were present on the side, and on the axial tip of the HVR.

## 4. Discussion

We and others have previously demonstrated that the chicken B-cell line, DT40, can be used to quantify the titer of IBDV field strains, and the titer of anti-IBDV serum neutralizing antibodies [15,16,17]. Here, we extend these observations by demonstrating that DT40 cells can be used to model IBDV antigenic drift and immune escape by passaging a classical genogroup A1 strain of IBDV, F52/70, in DT40 cells in the presence of sub-neutralizing concentrations of polyclonal serum antibodies raised against IBDV vaccine 2512. This simulated a situation that may occur in the field, where birds with poor neutralizing antibody titers elicited by sub-optimal vaccination may become infected with a classical field strain. Three escape mutants were generated, two that had the same mutations, D279Y and G281R at P10, and a third had mutations S251I and D279N. Furthermore, at P5, the D279Y mutation was detectable, but the G281R mutation was not, indicating the mutations arose with different kinetics.

Amino acid 251 is present in hydrophilic loop P-DE, and amino acids 279, and 281 are present in hydrophilic loop P-FG. The hydrophilic loops are neutralizing antibody sites [2,18], and mutations at these sites could, therefore, play a role in immune escape. Amino acid substitutions at these sites could alter antibody binding either directly, by being located in epitopes, or indirectly, by leading to conformational changes in epitopes that reduce the binding of neutralizing antibodies. However, we also noted the S251I mutation in the absence of immune selection pressure, suggesting it could play a role in DT40 cell entry. In the past, it has also been shown that a S251I mutation was involved in DT40- cell adaptation in the absence of immune selection pressure, in the classical virulent strain F52/70, and the very virulent strain UK661 [16]. Amino acid positions 279 and 281 have also been shown to be involved in DT40 cell adaptation in the absence of immune selection pressure: The classical virulent strain, GBF1, developed an N279Y/H mutation, the lab- adapted strain, Soroa, developed an N279D mutation [15,17], the US strain Del-E strain developed an N279H mutation, and the distinct strain M04/09 also developed an N279H mutation [16]. Moreover, during persistent infection of DT40 cells with IBDV strain Soroa, a mutation at position 281 was detected (G281L) [15]. It is, therefore, possible that the HVR mutations we identified could enhance immune escape by increasing the affinity of the virus for a cellular receptor or other protein involved in viral entry, thus promoting cellular entry in the face of neutralizing antibodies. Some of the mutations we identified have been previously described in field isolates: for example, a D279N mutation was identified in two vv genogroup A3 field isolates from Jordan [19], and the G281R mutation was found in a vv field isolate from Indonesia [19]. The presence of these amino acids in naturally occurring strains may be the result of selection pressures that increase virus fitness in the field. Of the mutations that we identified in the viruses passaged only in negative control serum, the A270E mutation is not located within a hydrophilic loo, but has previously been reported as being involved in DT40 cell adaptation in the vv strains OKYM and DV86 [17], and the S317R mutation is present in hydrophilic loop P-HI and has previously been described in Malaysian isolates [12]. Furthermore, a mixed population of 286T and 286I was present in the virus passaged in negative control serum at P5, but by P10, the amino acid was the same as P0 (286T), indicating this mutation did not become fixed in the virus population. Structural modeling revealed the mutations were on the side and axial tip of the HVR, consistent with previous reports of mutations in persistently infected DT40 cells [15], suggesting that IBDV may bind to DT40 cells in these regions.

Our study is not without limitations. We did not quantify antibody responses by ELISA or by a neutralization assay in DF-1 cells or chicken embryonic fibroblast (CEF) cells, which would have been beneficial, and we did not investigate cell-mediated immune responses. Additionally, although we titrated the P10 viruses in the presence of a 1:100 dilution of serum (Figure 2), we did not determine the end-point antibody dilutions for these viruses or compare the P10 viruses to P0. Moreover, while three independent escape mutants were generated, only one stock of virus was passaged in negative control serum. Furthermore, the whole genome was not sequenced, and it is possible that mutations outside of the HVR might also affect an immune escape. In the future, it would also be beneficial to apply next-generation sequencing technology, to detect minor variants in the viral populations, and the pathogenicity of the immune escape mutants should be characterized, as it is known that some of the HVR mutations can lead to alterations in virulence [3,5], although it should be noted that we did not identify any HVR mutations associated with a vv phenotype (222A, 242I, 256I, 294I, 299S) [12]. Furthermore, in order to confirm the extent to which the mutations we observed participate in immune escape, they could be introduced into a molecular clone of the virus by reverse genetics and the virus neutralization titer (VNT) quantified.

In summary, the antigenic changes that occur in the evolution of IBDV remain a challenge for effective disease control. Here, we modeled the antigenic drift of IBDV in vitro and identified amino acid substitutions in the HVR that resulted in escape from neutralizing antibodies. Our model could be used to characterize antigenic drift in different IBDV strains, and evaluate escape from serum antibody responses generated by different vaccination programs, in the future.

## Data Availability

Data is contained within the article.
